# A one-stop perineal clinic: our eleven-year experience

**DOI:** 10.1007/s00192-020-04405-2

**Published:** 2020-07-02

**Authors:** Osanna Yee Ki Wan, Annika Taithongchai, Susana I. Veiga, Abdul H. Sultan, Ranee Thakar

**Affiliations:** 1 Department of Obstetrics and Gynaecology, Croydon University Hospital, 530 London Road, London, CR7 7YE UK; 2 Department of Obstetrics and Gynaecology, Prince of Wales Hospital, The Chinese University of Hong Kong, Hong Kong, Hong Kong; 3 St George’s University of London, Cranmer Terrace, London, UK

**Keywords:** Anal incontinence, Female genital mutilation, Obstetric anal sphincter injuries, Perineal clinic, Perineal trauma, Perineal wound complications

## Abstract

**Introduction and hypothesis:**

The perineal clinic is a dedicated setting offering assessment for various childbirth-related presentations including obstetric anal sphincter injuries (OASIs), perineal wound complications, pelvic floor dysfunction and other conditions such as female genital mutilation(FGM). We describe the clinical presentation and outcomes of women from a tertiary perineal clinic based on data collected over an 11-year period.

**Methods:**

This is a retrospective observational study. A one-stop outpatient service was offered to all women who sustained OASIs (postnatally and antenatally in a subsequent pregnancy), perineal complications (within 16 weeks postpartum), FGM and/or peripartum symptoms of urinary/anal incontinence or prolapse. Assessment included history with validated questionnaires, examination and anal manometry and endoanal ultrasound when appropriate. Outcomes were compared among different grades of OASIs. Management of each type of presentation was reported with outcomes.

**Results:**

There were 3254 first attendance episodes between 2006 and 2016. The majority (58.1%) were for OASIs, followed by perineal wound complications. Compared to the lower grades, the higher grades of OASI were associated with poorer outcomes in terms of symptoms, investigations and complications. Women with OASIs had unrelated symptoms such as urinary incontinence, perineal pain and wound infections that needed further intervention. A high proportion(42%) of wound complications required further specialist management.

**Conclusion:**

We describe a dedicated, one-stop perineal clinic model for antenatal and postnatal women for management of perineal and pelvic floor disorders. This comprehensive and novel data will enable clinicians to better counsel women regarding of outcomes after OASI and focus training to minimize risks of morbidities.

## Introduction

Perineal trauma is the most common complication during vaginal delivery, occurring in 42% of women [1]. Obstetric anal sphincter injuries (OASIs) are the most severe form with potentially devastating effects on a mother’s quality of life [2, 3]. Up to 50% of women suffer from perineal pain and dyspareunia following OASIs, and these symptoms can last for years [4, 5]. There is also mounting evidence that the management and outcome of wound complications, persistent perineal pain or postpartum dyspareunia in those without OASIs remain unknown, although serious problems greatly affecting the physical and mental health of women have been reported [6].

These women frequently do not receive the dedicated care they need because of lack of knowledge in both healthcare professionals and women as well as poor care. The Royal College of Obstetricians and Gynaecologists (RCOG) have recommended that all women sustaining OASIs should have an assessment around 6 to 12 weeks postpartum by clinicians with a special interest in OASI where possible [7]. However, a survey of clinical leads of hospitals in the UK demonstrated that only 32% offered a dedicated outpatient clinic follow-up for women after OASIs [8]. Furthermore, postpartum women with perineal complications not related to OASIs should also be expected to have an opportunity for a similar assessment.

Women with female genital mutilation (FGM) have been reported to have poorer obstetric outcomes in both low-resource and resource-rich countries [9], with a contributing factor being lack of knowledge and experience of healthcare professionals. However, when appropriate antenatal counselling and management are available via a dedicated perineal clinic, obstetric outcomes showed no difference from those without FGM [10]. Peripartum women may also suffer from urinary incontinence, anal incontinence (AI) and prolapse symptoms affecting their quality of life, which may often be neglected because of lack of help available or the assumption that these symptoms are a “normal” consequence of childbirth, without appropriate investigation or assessment.

Various different models of perineal clinics have been reported. These include clinics led by consultant urogynaecologists [11–13], consultant obstetrician and gynaecologists [13, 14], with some triaged or assisted by specialized midwives/nurses [11, 12, 14, 15], and clinics led by a specialist midwife [16]. Formats of clinics include: one-stop clinics offering all necessary investigations and referrals [17, 18], a standard outpatient set-up where investigations are performed either at a later date [13–15] or prior to initial assessment [12], those where only limited investigations are available [15] or simple telephone follow-up [16]. The maximum number of patients reported in any series is up to 400 [14] and the longest duration of experience of perineal clinics reported was 4 years [11]. The targeted groups of patients seen also varied from those only focusing on postnatal OASIs [15] to including patients with perineal pain [11, 14] or AI outside the peripartum period of all ages [14], while some included AI only in antenatal or postnatal women [11, 13, 15]. Different management protocols have also been used for the management of OASI patients [19–21].

We described the clinical presentation and outcomes of women seen in our tertiary one-stop perineal clinic based on data collected over an 11-year period.

## Materials and methods

This is a retrospective observational study over an 11-year period between January 2006 to December 2016. A dedicated perineal clinic was established at Croydon University Hospital, London, UK, in 2002. All patient data were entered into a database prospectively. This weekly clinic provided a one-stop service for women, run by a consultant urogynaecologist with a concomitant perineal wound clinic, staffed by a trained specialist perineal midwife. Appropriate investigations as well as management of symptoms and counselling for future mode of delivery (MOD) when necessary were all offered at the same attendance [19]. Women are referred from within our institution, from local general practitioners or from surrounding hospitals. Referrals are accepted for women in both the postpartum period following OASIs and antepartum period with history of OASIs in a subsequent pregnancy, postpartum perineal wound complications such as infection, dehiscence, pain or dyspareunia, women with FGM either pregnant or outside of pregnancy, new symptoms of AI in the antenatal or postnatal period, complaints suggestive of a missed clinical diagnosis of OASIs or other symptoms of pelvic floor dysfunction in the peripartum period such as urinary incontinence, prolapse or sexual dysfunction. Other types of referrals include suspected genital tract fistula or other previous perianal/perineal surgery in pregnant women where MOD may be affected. The postpartum women were seen up to 16 weeks postnatally, after which they would be instead referred to the urogynaecology clinic or combined Pelvic Floor Clinic. Information regarding the clinic was sent to the women with their appointment letter.

A detailed history was obtained including demographic data (age, parity, ethnicity), MOD, obstetric details, degree of perineal tear and presence of vaginal, urinary or bowel symptoms. Perineal and anal sphincter trauma was classified using the Sultan classification [2, 7]. Severity of AI was assessed using the validated modified St. Mark’s incontinence score (SMIS), which ranged from 0 (no symptoms) to 24 (severe incontinence) [22]. Urinary incontinence was assessed using the validated International Consultation on Incontinence modular Questionnaire for Urinary Incontinence-Short Form (ICIQ-UI_SF), ranging from 0 (no symptoms) to 21 (severe incontinence) [17, 23]. Women underwent vaginal examination to assess wound integrity, size of the perineal body (cm) and pelvic floor muscle contraction using the Modified Oxford Scale [24]. Appropriate investigations such as anal manometry (AM) and endoanal ultrasound scan (EAUS) were performed in all women with history of OASIs irrespective of symptoms or grade of OASI as previously reported [18, 19] and when clinically indicated. Defects in the sphincter muscle were defined as ≥ 30° disruption (equivalent to > 1 h on the clock face) in the external anal sphincter (EAS) at the deep, superficial and/or subcutaneous levels or the internal anal sphincter (IAS) as previously described [22].

### Patient management

Perineal pain and/or dyspareunia, urinary incontinence and pelvic organ prolapse were managed as per structured local evidence-based or best practice protocols (available on www.perineum.net). Women with a history of OASIs were counselled regarding subsequent MOD, based on presence of symptoms and investigation findings as previously reported [19], as well as ensuring they were practising pelvic floor exercises (PFE). Perineal wound infection was diagnosed on clinical examination as evidenced by local perineal tenderness, erythaema, exudate, odour and oedema, with or without pyrexia. Wound dehiscence was diagnosed when there was gaping of the perineal wound (> 0.5 cm). Wound swabs were taken for culture and sensitivities and oral antibiotics commenced (local protocol as agreed with microbiology being amoxicillin/clavulanic acid), loose sutures removed, perineal washout performed if necessary and perineal hygiene advice given). In cases of systemic infection, intravenous antibiotics were recommended with twice daily perineal washout. Women with wound dehiscence were counselled regarding the options of wound re-suturing or healing by secondary intention given that there is a lack of evidence of best practice for management of wound dehiscence [25]. Wound infections were initially reviewed weekly every 2–3 weeks and for wound dehiscence review would be every 2 weeks once the infection is controlled.

Women who had undergone FGM were questioned regarding associated urinary, bowel, sexual and infective symptoms. Pregnant women requiring de-infibulation were advised to have it done in the second trimester. Other FGM complications such as cysts and de-infibulation in non-pregnant women would be managed accordingly.

Women with perineal pain or dyspareunia were assessed for abscesses or fistulae by vaginal and/or rectal examination and EAUS or perineal ultrasound. Management includes examination under anaesthesia, antibiotics, incision and drainage of the abscess or removal of irritant undissolved suture materials as necessary. Persisting granulation tissue was treated with silver nitrate application.

In cases of persistent perineal or scar tenderness, perineal massage with topical 5% lidocaine ointment was advised. If there was associated dyspareunia, the women were asked to apply it about 30 min prior to coitus. If the problem still persisted, a cocktail of 10 ml 0.5% bupivacaine, 1500 IU hyaluronidase and 40 mg Depo-Medrone (methylprednisolone acetate) solution was injected to the site of maximal tenderness. A second or third injection could be repeated after a minimum of 6 weeks. Vaginal dilators, psychosexual counselling, myofascial release and/ or perineoplasty were recommended when indicated. Women with an anal fissure were given advice with regard to relieving constipation by having a high fibre diet along with fibre supplements such as Fybogel (Ispaghula Husk) and oral Lactulose in combination with topical anal application of lidocaine ointment prior to defecation and perineal hygiene advice. Local application of glycerine trinitrate 0.4% ointment was also prescribed to relax IAS spasm, frequently associated with anal fissures.

### Ethics approval

All investigations were part of routine clinical practice, and therefore ethical approval was not required.

### Statistical analysis

The data were analysed using IBM SPSS statistics version 22. Descriptive analysis was used to study the demographics, symptoms of AI, urinary incontinence and their respective SMIS and ICIQ-UI scores, and the incidence of wound complications in women with OASIs. The Fisher’s exact test, chi-squared test, Student’s t-test and Mann-Whitney U test were used for statistical comparisons. *p* < 0.05 was taken as statistical significance.

## Results

There were 3254 first attendances between January 2006 and December 2016. The mean age was 30.3 ± 5.5 years, and the mean body mass index was 25.1 ± 4.7 kg/m^2^. The median parity was 1 [interquartile range (IQR) 1, 2]. The median follow-up was 10 weeks (IQR 5, 17) after delivery.

The women were of mixed ethnicity, including 40.2% White, 20.6% Black African, 10.6% Asian Indian, 15.0% other Asian and 13.6% other ethnicity. The majority (70%) were patients from our institution, 20% were referred by the general practitioner, and 10% were tertiary referrals from other hospitals. Most (58.9%) were seen during the postpartum period, 35.6% were seen during the antenatal period while the remaining 5.5% were unrelated to pregnancy.

The primary reasons for referral to the perineal clinic are listed in Table 1.

**Table 1:** Primary reasons for referral to perineal clinic

Reason for referral	Number of women (%) (*N* = 3254)
Obstetric anal sphincter injury (OASI)	1892 (58.1)
OASI managed at 3 months postnatally	
Third-degree tear	1085
*Third-degree tear 3a*	*422*
*Third-degree tear 3b*	*420*
*Third-degree tear 3c*	*160*
*Unclassified third-degree tear*	*83*
Fourth-degree tear	62
Rectal buttonhole tear	3
OASI managed antenatally in a subsequent pregnancy	742
Perineal complications	765 (23.5)
Wound infection	236
Wound dehiscence	209
Perineal pain/dyspareunia	225
Other perineal wound problems such as haematoma/swelling/migrating stitches/labial adhesions	66
Perineal masses such as varicosities, Bartholin’s cyst	16
Extensive perineal tear (not OASIS) requiring debriefing	13
Female genital mutilation (FGM)	318 (9.8)
FGM type 1	93
FGM type 2	138
FGM type 3	35
FGM type 4	3
Patient declined examination/unclassified FGM	8
No definite FGM when examined	41
Peripartum pelvic floor problem	74 (2.3)
Urinary incontinence	43
Urogenital prolapse	30
Voiding dysfunction	1
Bowel problem (without a definite history of OASI)	136 (4.2)
Anal incontinence	90
*Faecal incontinence*	*69*
*Flatal incontinence*	*21*
Faecal urgency	44
Others: constipation, per-rectal bleeding	2
Referred for suspected missed or undiagnosed OASIS after delivery	42 (1.3)
Due to deficient perineum in postpartum examination by GP	6
Delivery with uncertain degree of perineal trauma or patient worried about third-degree tear	36
Referred for advice on mode of delivery	27 (0.8)
Perianal disease/ulcerative colitis/fistula	4
History of anal surgery, e.g., sphincterotomy, fissurectomy	7
History of fistula repair	4
History of perineal refashioning	10
Vaginal septum/vaginal hymen ring	2

FGM = female genital mutilation, GP = general practitioner, OASI = obstetric anal sphincter injury

### OASI

Urinary incontinence was the most common symptom, with up to 16.5% having a mean ICIQ-UI score of 1.9 ± 3.8, followed by flatal incontinence in 11.8%, with a mean SMIS of 1.9 ± 3.7 (Table 2). Overall incidence of perineal pain and dyspareunia in women with OASI was 6.7% and 2.4%, respectively. Treatment options included application of silver nitrate for granulation tissue and perineal massage (15.6%) for perineal pain with two requiring a steroid cocktail injection for persisting perineal pain. Wound infection and dehiscence were found in 2.7% and 3.3% in women with OASI, respectively. Ten women (1.0%) had re-suturing of their perineal wound for dehiscence and 11 (1.0%) women underwent a secondary sphincter repair (one 3a, two 3b, five 3c tears, one unspecified third-degree and two 4th-degree tears). Urogenital prolapse was found in 1.0%, with five apical and anterior compartment prolapses, two anterior and posterior compartment prolapses, two posterior compartment prolapses only and one apical compartment prolapse only. There were eight women with fistula: one perineorectal, two perineovaginal, three rectovaginal (1 with a concomitant perineovaginal fistula and 1 after a rectal button-hole tear) and another two trans-sphincteric, all requiring surgical intervention.

**Table 2:** Clinical outcomes of obstetric anal sphincter injuries at 3 months postnatally (excluding the 3 patients with rectal buttonhole tears)

Number of women (%)	Total (*N* = 1147)	3a tear (*n* = 422)	3b tear (*n* = 420)	3c tear (*n* = 160)	Ungraded third-degree tear grade (*n* = 83)	Fourth0degree tear (*n* = 62)	*p* value^a^ OR, 95% CI	*p* value^b^ OR, 95% CI	*p* value^c^ OR, 95% CI
^d^Flatal incontinence (%)	135 (11.8)	32 (7.6)	61 (14.5)	13 (8.1)	13 (15.9)	16 (25.8)	0.01* OR = 1.8, 1.2–2.7	0.27	< 0.01* OR = 2.8, 1.6–5.1
^d^Faecal incontinence (%)	45 (3.9)	7 (1.7)	13 (3.1)	12 (7.5)	8 (9.8)	5 (8.1)	0.02* OR = 2.7, 1.1–6.2	0.001* OR = 3.3, 1.6–7.0	0.08
^d^Faecal urgency (%)	90 (7.9)	21 (5.0)	31 (7.4)	20 (12.5)	13 (15.9%)	5 (8.1)	0.02* OR = 1.8, 1.1–3.1	0.01* OR = 2.2, 1.3–3.8	0.92
Mean St. Mark’s incontinence score ± SD	1.9 ± 3.7	1.1 ± 2.6	1.8 ± 3.5	2.5 ± 4.5	4.3 ± 4.9	1.8 ± 3.6	< 0.01	< 0.01*	< 0.01*
Urinary incontinence (%)	189 (16.5)	70 (16.6)	68 (16.2)	22 (13.8)	18 (22.0)	11 (17.7)	0.40	0.46	0.96
Mean ICIQ-UI score	1.9 ± 3.8	1.8 ± 3.7	1.5 ± 3.2	1.6 ± 3.7	5.1 ± 6.5	2.0 ± 4.2	0.33	0.84	0.81
Wound infection (%)	31 (2.7)	6 (1.4)	13 (3.1)	3 (1.9)	6 (7.3)	3 (4.8)	0.16	0.76	0.23
Wound dehiscence (%)	38 (3.3)	5 (1.2)	15 (3.6)	4 (2.5)	9 (11.0)	5 (8.1)	0.71	0.64	0.18
Dyspareunia (%)	27 (2.4)	9 (2.1)	7 (1.7)	3 (1.9)	6 (7.3)	2 (3.2)	0.63	0.98	0.65
Perineal pain (%)	77 (6.7)	21 (5.0)	23 (3.5)	13 (8.1)	15 (18.3)	5 (8.1)	0.42	0.15	0.64
Vaginal adhesion/perineal sinus (%)	13 (1.1)	3 (0.7)	6 (1.4)	1 (0.6)	3 (3.7)	0	0.44	0.61	1.0
Granulation tissue (%)	25 (2.2)	5 (1.2)	8 (1.9)	3 (1.9)	7 (8.5)	2 (3.2)	0.38	0.76	0.67
Stitch migration (%)	4 (0.3)	1 (0.2)	0	0	0	3 (4.8)	0.24	0.66	< 0.01* OR: 55.1, 5.6–538.0
Fistula (%)	7 (0.06)	0	0	1 (0.63)	3 (3.66)	3 (3.66)	0.14	0.07	0.01* OR: 6.0, 1.2–30.3
Urogenital prolapse (%)	10 (0.9)	3 (0.7)	1 (0.2)	4 (2.5)	2 (2.4)	0	0.80	0.008* OR = 5.4, 1.3–21.7	1.0
Anal fissure (%)	29 (2.5)	9 (2.1)	13 (3.1)	4 (2.5)	3 (3.7)	0	0.44	0.93	0.4
Mean size of perineal body	2.5 ± 2.0	2.5 ± 0.7	2.5 ± 0.7	2.5 ± 0.5	2.4 ± 0.6	2.0 ± 0.7	1.0	1.0	< 0.01*
Anal manometry results									
Mean resting pressure (mmHg) ± SD	50.3 ± 14.3	53.1 ± 13.5	50.1 ± 13.6	48.0 ± 16.4	47.3 ± 16.1	40.3 ± 10.4	< 0.01*	0.007*	< 0.01*
Maximum squeeze pressure (mmHg) ± SD	94.6 ± 22.0	93.4 ± 27.3	85.1 ± 26.2	83.6 ± 29.4	81.0 ± 21.9	68.1 ± 19.0	< 0.01*	0.04*	< 0.01*
Maximum cough reflex pressure (mmHg) ± SD	73.6 ± 22.5	79.2 ± 20.8	71.6 ± 21.7	72.8 ± 25.8	71.0 ± 25.7	60.1 ± 14.1	< 0.01*	0.41	< 0.01*
Maximum incremental rise ± SD	36.6 ± 21.0	40.4 ± 22.7	35.0 ± 20.0	35.5 ± 21.0	33.7 ± 14.9	28.3 ± 15.4	< 0.01*	0.28	< 0.01*
Mean anal length (cm) ± SD	2.4 ± 0.5	2.4 ± 0.5	2.3 ± 0.5	2.3 ± 0.6	2.2 ± 0.5	2.3 ± 0.6	0.004*	0.43	0.14
Endoanal ultrasound results									
Defect in EAS (%)	213 (18.6)	36 (8.6)	88 (21.0)	43 (26.9)	16 (19.3)	30 (48.4)	< 0.01* OR = 3.1, 2.1–4.6	< 0.01* OR = 2.2, 1.5–3.3	< 0.01* OR: 4.9, 2.9–8.4
Defect in IAS (%)	196(17.0)	23 (5.5)	67 (16.0)	50 (31.3)	14 (16.9)	42 (67.7)	< 0.01* OR = 4.4, 2.7–7.0	< 0.01* OR = 3.9, 2.6–5.9	< 0.01* OR: 14.5, 8.0–26.1

95% CI = 95% confidence interval; EAS = external anal sphincter; IAS = internal anal sphincter; ICIQ-UI = International Consultation on Incontinence modular Questionnaire for Urinary Incontinence; OR = odds ratio; SD = standard deviation

*Statistically significant *p* value

a: comparing between 3a and 3b/c tear grade

b: comparing between 3a/b and 3c tear grade

c: comparing between overall third- and fourth-degree tear

As demonstrated in Tables 2 and 3, compared to women who sustained a third-degree tear, women with a fourth-degree tear had almost three times higher risk of having flatal incontinence (*p* < 0.01), higher mean SMIS (3.9 ± 5.7 vs. 1.8 ± 3.6, *p* < 0.01), more complications such as migrated sutures [OR (odds ratio) = 55, 95% CI (confidence interval) 5.6–538.0] and more referrals for further management to the joint pelvic floor clinic for colorectal input. Furthermore, women with clinically diagnosed IAS injury, i.e., 3c tear, were 2.2 times more likely to have faecal urgency, 3.3 times more faecal incontinence, 5.4 times more likely to have urogenital prolapse, had lower AM pressures and were more likely to have persistent EAS (2.0 times) and IAS (4.0 times) defects. Moreover, when comparing individual compartment prolapse, there no statistical differences were found between different degrees of tear. OASI did not have a statistically significant association with urogenital prolapse after adjustment by foetal size and mode of delivery in multivariate analysis. No differences were found among different grades of third-degree tears for other wound outcomes (Tables 2 and 3).

**Table 3:** Treatment or procedures required for complications in women with obstetric anal sphincter injuries (excluding the 3 women with a rectal buttonhole tear)

Number of women (%)	Total (N = 1147)	3a tear (n = 422)	3b tear (n = 420)	3c tear (n = 160)	Ungraded 3rd-degree tear (n = 83)	Fourth-degree tear (n = 62)	*p*-value^a^ OR, 95% CI	*p*-value^b^ OR, 95% CI	*p*-value^c^ OR, 95% CI
^d^Wound re-suturing	10 (0.9)	2 (0.5)	5 (1.2)	1 (0.6)	1 (1.2)	1 (1.6)	0.33	0.79	0.42
^d^Secondary sphincter repair	11 (1.0)	1 (0.2)	2 (0.5)	5 (3.1)	1 (1.2)	2 (3.2)	0.09	< 0.01* OR = 9.0, 2.1–38.2	0.11
^d^Lidocaine gel for perineal pain	23 (2.0)	10 (2.4)	5 (1.2)	4 (2.5)	4 (4.9%)	0	0.34	0.54	0.63
^d^Cocktail injection	2	0	1	0	1	0	0.58	0.84	0.90
^d^Silver nitrate for granulation tissue	21 (1.8)	3 (0.7)	9 (2.1)	2 (1.3)	6 (7.3%)	1 (1.6)	0.12	0.86	1.0
^d^Vaginal dilators	5 (0.4)	1 (0.2)	2 (0.5)	0	1 (1.2)	1 (1.6)	0.76	0.45	0.24
^d^Scar re-fashioning	5 (0.4)	2 (0.5)	3 (0.7)	0	0	0	0.93	0.33	1.0

95% CI = 95% confidence interval; OR = odds ratio

*Statistically significant *p*-value

a: comparing between 3a and 3b/c tear grade

b: comparing between 3a/b and 3c tear grade

c: comparing between overall third- and fourth-degree tear

d: chi-square test and Fisher exact tests are used

There were 742 women seen antenatally in a subsequent pregnancy. Around 21.6% had symptoms of AI with the mean SMIS being 1.9 ± 4.6. Persistent EAS and IAS defects were found in 21.8% and 21.0%, respectively. Of these women, 11.3% were advised for caesarean section, and 78.0% were advised that there was no contraindication to vaginal delivery, with a low threshold for episiotomy. The remaining were either undecided during the consultation or the decision was unknown because it was dependent on other obstetric factors such as foetal size or placental location. The obstetric outcomes for women following their subsequent pregnancies have been reported previously [19].

### Postnatal perineal wound complications

Perineal complications were the second most common reason for attendance to the perineal clinic, namely wound infection (30.9%), perineal pain (29.5%), wound dehiscence (27.4%), other perineal masses, stitch migration or haematoma formation (12.3%). More than 42% of them required further intervention, including 64 women (8.4%) who underwent secondary re-suturing of the perineal wound, 119 (15.6%) silver nitrate application, 12 (1.6%) suture removal, 41 (5.4%) scar refashioning, 58 (7.6%) and 17 (2.2%) local anaesthetic and steroid cocktail injections for pain relief, respectively, and 13 (1.7%) vaginal dilators.

### Female genital mutilation

The most common type of FGM presentation was type 2 (partial or total excision of the clitoris and labia minora with or without removal of the labia majora): 242 (76.1%) were seen antenatally, 62 (19.5%) postnatally, and 14 outside of the peripartum period. In total, 17 women underwent surgical treatment, including 7 de-infibulations performed antenatally, 4 de-infubulations intrapartum, 1 postnatal perineal reconstruction and a further 5 de-infibulations in non-pregnant women.

### New bowel symptoms without OASIs

Most of these women (134) had symptoms of AI; one had constipation and the other per rectal bleeding. The majority (80.2%) were referred postnatally. Their mean SMIS was 10.0 ± 6.0. All women presenting with symptoms of AI and no history of OASI were offered AM and EAUS for assessment. EAS and IAS defects were found in 38 (36.8%) and 27 (19.5%) women, respectively; 40 (29.4%) women were diagnosed to have had clinically missed OASIs at the time of delivery and were managed as previously described [18]. Functional anal length, mean maximum resting pressure, mean maximum squeeze pressure and mean maximum cough reflex pressure were 1.7 ± 1.1 cm, 38.0 ± 24.4 mmHg, 77.0 ± 26.5 mmHg and 69.9 ± 23.1 mmHg, respectively. All were taught PFE, 25 (18.8%) were referred for biofeedback for bowel symptoms, 6 (4.5%) required loperamide for symptom control, 6 (4.5%) were offered secondary repair as they had completed their family and 1 (0.8%) had sacral nerve modulation.

### Peripartum urinary incontinence

The mean ICIQ-UI score was 11.6 ± 4.9 for those presenting with a primary complaint of UI. Up to 95.3% were advised to practise PFE, 20.9% underwent bladder retraining, 7.0% had biofeedback, and 4.7% were prescribed anti-cholinergics. About one third had further follow-up with the urogynaecology nurse specialist and the remainder did not require further referral.

### Peripartum pelvic organ prolapse

A clinical diagnosis of pelvic organ prolapse was confirmed in 22 (73.3%) with 52.9% suffering from stage II and the remaining stage I pelvic organ prolapse by the Pelvic Organ Prolapse Quantifications System. Four women chose pessary insertion and two out of the four eventually opted for a surgical repair: one sacrohysteropexy and one anterior and posterior pelvic floor repair.

## Discussion

We describe the clinical presentation and outcome of 3254 women seen in a dedicated one-stop perineal clinic based on data collected over an 11-year period. As far as we know, this is the largest reported series of patients in the literature attending the first dedicated one-stop perineal clinic with EAUS and AM. The most common reason for referral was OASIs, with the second being perineal wound complications, with a high proportion requiring further management. Women who sustained 3b or more severe OASIs had significantly higher risks of symptoms of AI, poorer performance on AM, persistent sphincter defects on EAUS and more complications such as fistula formation or need for a secondary sphincter repair.

Although many published studies evaluate AI after OASIs [2, 4, 5, 26], other associated complications, outcomes and management have not been described [22, 27]. The large number in our cohort provided us a unique opportunity to report this. We found that the degrees or grades of OASIs did not differ in the incidence of wound infection, dehiscence or pain. However, a significant proportion of women who sustained OASIs required further postnatal treatment for management of pain, granulation tissue, wound infection or dehiscence. This demonstrates the importance of offering these women the opportunity to be examined postnatally and enquiring about symptoms besides the specific assessment of bowel symptoms and sphincter integrity.

A significantly shorter mean size of the perineal body was found in women who sustained fourth-degree tears compared to those with third-degree tears (2.0 ± 0.7 vs. 2.5 ± 1.3, *p* < 0.01). This supports the importance of examining the perineal body during postpartum care, especially in cases of possible missed tears [18]. The reconstruction of the perineal body muscles is particularly important for supporting the sphincter repair as a short and deficient perineum can pose higher risks of OASI recurrence in future deliveries [3].

We found women with a 3c tear to be more likely to develop a fistula (OR = 6.0, 95% CI = 1.2–30.3) and undergo a secondary anal sphincter repair (OR = 9.0, 2.1–38.2), highlighting the importance of training in making the correct diagnosis of the full extent of injury as well as paying particular attention to adequate primary IAS repair [2, 22]. In addition, they are 5.4 times more likely to develop symptoms of urogenital prolapse, highlighting the importance of encouraging women to perform PFE.

In keeping with a previous study [17], we found that urinary incontinence was the most common concomitant symptom with OASIs. We acknowledge that this is a very select group of women with OASIs without a comparative control group and this could be a common transitory postnatal change. However, it does highlight the importance of advocating PFE to these women. In women without OASIs who presented with urinary incontinence, although having high ICIQ-UI scores, few required ongoing management after PFE indicating that implementing simple conservative treatments is often all that is necessary.

Reported rates of wound infection and dehiscence in women with perineal trauma vary between 0.1%–23.6% and 0.21%–24.6%, respectively [6]. Specific data on wound complications in OASIs are limited [28]. We found the rate after OASI to be on the lower side (2.7% infection and 3.3% dehiscence, respectively). This could be due to technique but also women with OASIs are prescribed antibiotics prophylactically at the time of repair [7]. Our local protocol advocates oral antibiotics for 3 days in addition to intra-operative intravenous antibiotics.

The second most common reason for attendance was perineal wound complications with a very high proportion requiring specific treatment for their symptoms and management options, which may not necessarily be available in other settings. Without a dedicated clinic, it is possible that the vast majority would have persistent symptoms without accessing help, but just suffering in silence. There are no internationally recognized guidelines on management of symptoms such as perineal pain, dyspareunia or wound dehiscence but we have developed protocols that have provided excellent standardized treatment (www.perineum.net). We advocated the use of short-term prophylactic antibiotics after repair of complex perineal tears but not simple tears. A recently published large multicentre randomized trial found that one prophylactic dose of antibiotic in women undergoing operative vaginal birth significantly reduced the rate of perineal infection [29].

The establishment of an open-access dedicated one-stop clinic enabled provision of evidence-based standardized care by experienced professionals. These clinic data show the multiple benefits to women of this type of service: the opportunity to explore the circumstances under which their perineal injury occurred, understand the extent of trauma and in many cases provide simple reassurance. A duty of candour and giving consistent advice to women is important from a medicolegal perspective particularly as this is an area of obstetrics where there is increasing litigation. It is also an arena where taboo topics such as FGM could be openly discussed and managed by experienced professionals with little to no barriers. This is particularly important for antenatal patients as it is a crucial time to identify these women and arrange appropriate treatment options.

The one-stop model avoids multiple visits to the hospital, an important consideration in postpartum women. We found that combined AM and EAUS performed by the same physician at one visit optimizes the management plan by correlating with symptoms enabling a holistic approach to women with clinically recognized and unrecognized OASIs [18]. With improved clinical detection of OASIs in recent years due to improved training there is also a risk of overdiagnosis, reported in one large series to be 7% [30].

We acknowledge the limitations of our article. We were unable to report data on women who have presented to other hospitals with their complications. While we were unable to report the incidence of wound complications in women with OASIs, women with other perineal trauma were not followed up routinely in our setup; therefore, establishing the total percentage of wound complications for all women was not possible. We also do not have a control group for comparison of outcomes and a set follow-up period to report on the natural history of these conditions. Some conditions might self-resolve over time, regardless of the intervention. In addition, ours is a tertiary referral centre and therefore may provide an over-representation of symptoms. We did not have patient satisfaction data over this period. In recent times, there has been an increased awareness of postpartum complications among healthcare professionals and patients. Hence, we could have witnessed higher rates of referrals. We appreciate that our findings and opinions are based on the experience of a single centre and our study population was heterogeneous, possibly making our results generalizable. However, the data were collected prospectively, limiting recall bias. Other strengths include the use of validated questionnaires, objective validated investigative tools and the use of standardized management protocols.

## Conclusion

There is undoubtedly considerable attention directed toward antenatal women with regard to foetal and maternal conditions. By comparison, there is a relative disparity in postpartum care, particularly related to the pelvic floor and perineum. This article demonstrates the utility, justification and benefits of a dedicated, one-stop, perineal clinic for postpartum women who have specific problems related to their perineum, anal sphincters, vagina or pelvic floor. Apart from women who have sustained OASIs there are postpartum women who suffer bowel, bladder, prolapse and sexual problems and many of these women suffer in silence. In a system of universal healthcare and equality, one could argue that if a local institute is unable to provide the above model of care, then these patients should ideally be referred to a hospital able to provide the necessary investigations and management. These comprehensive and novel data enable clinicians to better counsel women regarding outcomes after OASI and other perineal problems, focus training to minimize risks of morbidities, justify the establishment of one-stop perineal clinics and enhance data collection for audit, research and development.
